# GluA1 Phosphorylation Alters Evoked Firing Pattern *In Vivo*

**DOI:** 10.1155/2012/286215

**Published:** 2012-04-09

**Authors:** Balázs Barkóczi, Gábor Juhász, Robert G. Averkin, Imre Vörös, Petra Vertes, Botond Penke, Viktor Szegedi

**Affiliations:** ^1^Bay Zoltán Foundation for Applied Research, BAYGEN, Közép Fasor 41, Szeged 6727, Hungary; ^2^Department of Medical Chemistry, University of Szeged, Szeged, Hungary; ^3^Research Group for Cortical Microcircuits of the Hungarian Academy of Sciences, University of Szeged, Közép Fasor 52, Szeged 6726, Hungary; ^4^Department of Physiology, Anatomy and Neuroscience, University of Szeged, Közép Fasor 52, Szeged 6726, Hungary; ^5^St John's Collage, University of Oxford, Oxford, UK; ^6^Behavioural & Clinical Neuroscience Institute, University of Cambridge, Cambridge, UK

## Abstract

AMPA and NMDA receptors convey fast synaptic transmission in the CNS. Their relative contribution to synaptic output and phosphorylation state regulate synaptic plasticity. The AMPA receptor subunit GluA1 is central in synaptic plasticity. Phosphorylation of GluA1 regulates channel properties and trafficking. The firing rate averaged over several hundred ms is used to monitor cellular input. However, plasticity requires the timing of spiking within a few ms; therefore, it is important to understand how phosphorylation governs these events. Here, we investigate whether the GluA1 phosphorylation (p-GluA1) alters the spiking patterns of CA1 cells *in vivo*. The antidepressant Tianeptine was used for inducing p-GluA1, which resulted in enhanced AMPA-evoked spiking. By comparing the spiking patterns of AMPA-evoked activity with matched firing rates, we show that the spike-trains after Tianeptine application show characteristic features, distinguishing from spike-trains triggered by strong AMPA stimulation. The interspike-interval distributions are different between the two groups, suggesting that neuronal output may differ when new inputs are activated compared to increasing the gain of previously activated receptors. Furthermore, we also show that NMDA evokes spiking with different patterns to AMPA spike-trains. These results support the role of the modulation of NMDAR/AMPAR ratio and p-GluA1 in plasticity and temporal coding.

## 1. Introduction

Alpha-amino-3-hydroxyl-5-methyl-4-isoxazole-propionate (AMPA) and N-methyl-D-aspartic acid (NMDA) type ionotropic glutamate receptors mediate fast neuronal excitatory transmission and play key roles in various forms of hippocampal synaptic plasticity. AMPARs comprise four subunits—GluA1, GluA2, GluA3, and GluA4 (or GluR1-4)—that combine to form tetramers. GluA1 is critical in several forms of hippocampal synaptic plasticity, correlates of learning, and memory. Mutant mice lacking the GluA1 subunit display impairments of the initial component of long-term potentiation (LTP) at CA1 in the hippocampus [1–4]. Moreover, these animals exhibit a robust spatial deficit in working memory [5–7]. Emerging evidence shows that as a rapid and short-term mechanism, dynamic protein phosphorylation directly modulates the electrophysiological properties of cells, as well as the trafficking/clustering and synthesis of AMPA receptors. The phosphorylation state of GluA1 has been demonstrated to be actively involved in regulating synaptic plasticity and, consequently, learning and memory processes. Phosphorylation of serine 831 and dephosphorylation of serine 845 are differentially involved in the modulation of LTP and LTD, respectively. The former serine is specifically phosphorylated by protein kinase C (PKC) and Ca^2+^/calmodulin-dependent protein kinase II (CaMKII) [8, 9], whereas the latter serine is specifically phosphorylated by protein kinase A (PKA).

It is widely accepted that neuronal discharge rates, averaged over several hundred milliseconds to several seconds, reflect the properties of cellular input, with more intense incoming stimuli resulting in enhanced firing rates. However, increasing the excitatory drive arriving onto a neuron may be achieved by either increasing the strength of excitation via the activation of new inputs or, alternatively, by modifying the excitatory inputs already in use, mainly through phosphorylation. In this paper, we test the hypothesis that these two scenarios may result in different spiking patterns. Precise timing of spiking activity of pre- and postsynaptic neurons in the millisecond range is required for modulating synaptic strength, a phenomenon referred to as spike timing synaptic plasticity. Moreover, the temporal relationship of spikes is also critical for information transfer [5, 10–13]. It is therefore crucial to understand the physiological mechanisms that can generate precise spike-timing *in vivo*.

Tianeptine, an antidepressant with distinct pharmacology to the tricyclic antidepressant agents, has been shown to increase the phosphorylation level of GluA1 on both the CaMKII and PKA site, which in turn renders the cell more sensitive to AMPA [14]. We have shown previously that intraperitoneal tianeptine administration results in an increased AMPA-evoked spiking rate *in vivo* [15]. Here, we investigate the impact of enhanced AMPA sensitivity on the AMPA-evoked spiking pattern of CA1 neurons, and we compare NMDA and AMPA-evoked spike trains. Using frequency of spike discharge as an indicator of somatic depolarization, we show that similar levels of excitation can be achieved by either raising AMPA sensitivity or increasing the concentration of the agonist. Despite the similar firing rates, the interspike interval distributions are significantly different among the two groups, showing that neuronal output may differ when new inputs are activated compared to increasing the gain of previously activated inputs.

## 2. Experimental Procedures

### 2.1. Animal Care and Handling

The animals were kept, and the experiments were conducted in conformity with Council Directive 86/609/EEC, the Hungarian Act of Animal Care and Experimentation (1998, XXVIII), and local regulations for the care and use of animals for research.

### 2.2. *In Vivo* Microiontophoresis and Single-Unit Electrophysiology

Extracellular single-unit recordings were taken in chloral-hydrate-anesthetized (4 g/kg initial dose, i.p., supplemental doses as required) male Wistar rats weighing between 300 and 350 g. The head of the animal was mounted in a stereotaxic frame, the skull was opened above the hippocampus (a-p: −3.8 mm from bregma; lat: ±2 mm either side from the midline), and the *dura mater* was carefully removed.

### 2.3. Extracellular Recordings and Iontophoresis

 Single-unit activity was extracellularly recorded by means of a low impedance (<1 M*Ω*) 7 *μ*m carbon fiber-containing microelectrode from the hippocampus between the depths of 2 to 3 mm, and drugs were delivered from the surrounding outer barrels. The action potentials were amplified with an ExAmp-20KB extracellular amplifier (Kation Scientific, Minneapolis, MN) and monitored with an oscilloscope. Filter bandpass frequencies were 300 to 8000 Hz. The amplified signals were sampled and digitalized at 50 kHz frequency. Spikes were sorted using the Spike2 software package (CED, Cambridge, UK). Iontophoretic drug delivery was performed by iontophoretic pumps (Minion-16 and BAB-350, Kation Scientific). A multibarrel electrode affixed to the recording electrode was used for the iontophoretic ejection of the following drugs: 100 mM NMDA Na^+^ salt in 100 mM NaCl (pH = 8) or 10 mM AMPA (pH = 8). NMDA was ejected at negative iontophoretic current ranging from 5 to 100 nA every minute for 5 sec. AMPA was ejected every 90 sec at negative iontophoretic current ranging from 2 to 100 nA. A retaining current of opposite direction between 2 and 21 nA was used. Tianeptine was administered as a single bolus intraperitoneal injection at a dose of 20 mg/kg.

### 2.4. Data Analysis

Spike timing was converted into numerical data at 30 *μ*s resolution. Spike trains were characterized by their interspike interval (ISI) series and compared. First, we compared the distribution of intervals for each pair of spike trains, assuming the measured intervals to be samples of mutually independent random variables. This allowed the use of Kolmogorov-Smirnov tests, which compare every characteristic of the underlying probability distributions, thus requiring no further assumptions. We then charted the maximal confidence intervals of agreement between the pairs. Data were pooled, and means ± SEM of percentage values were calculated. PSTH and ISI probability profiles were evaluated by using ANOVA with post hoc Dunnett's test. A *P* value of ≤0.05 was considered as significant difference in all cases.

In order to investigate the temporal evolution of spiking activity, the time course of the spiking activities was normalized, so that the first spike was taken as time 0, while the last spike of the train was taken as 1. ISIs were plotted against this normalized time. To fit smooth curves to the spike trains, first we transformed them by taking their negative logarithms, which corresponds to a spiking intensity value. To compare the actual dynamics of the spike trains, we noted that ISI logarithms fit convincingly to quadratic polynomials, that is, the ISI series itself was fitted with the dilated Gaussian exp⁡⁡(*a*∗(*x*−*a*0)^2^ + *c*) equation using a standard linear. From this, we produced basic statistics of the coefficients for each group of spike trains.

## 3. Results

**Figure 1:**
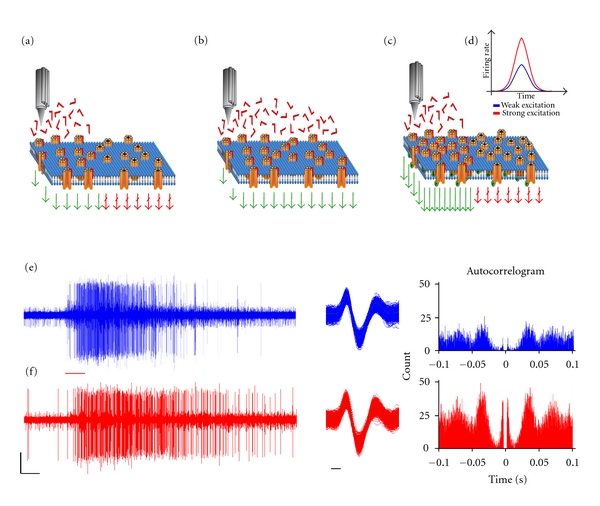
Schematic outline of the experiment. CA1 cells were excited by a small (a) or large (b) dose of excitatory compound (AMPA or NMDA). In a second set of experiments, AMPA receptor phosphorylation (GluA1 subunit), which might lead to increased AMPA receptor surface expression and subsequent rise in AMPA-evoked firing rate, was induced by an intraperitoneal tianeptine injection (c). Strong excitations triggered larger firing rates than weak excitations (d). Representative spike trains, superimposed spikes, and autocorrelograms of the same unit before (e) and 15 min after tianeptine injection (f). Red line marks the ejection event. Scale bars are 50 *μ*V and 1 sec and 0.1 ms.

Tianeptine has been shown to increase the phosphorylation level of AMPA receptor subunit GluA1 at Ser 831 and Ser 845 [14, 16]. This effect results in increased AMPA-evoked spiking activity *in vivo* after intraperitoneal (i.p.) tianeptine application. We hypothesized that this effect also leads to alternation of the spiking pattern evoked by AMPA. To address this point, we have recorded spike trains evoked by weak and strong AMPA stimulation and compared the data to AMPA-evoked spike trains recorded before and after i.p. tianeptine application. Furthermore, we compared those with NMDA-evoked spiking activity. Ejection currents were unaltered before and after tianeptine within an experiment. The outline of the experimental setup is indicated in Figure 1. A total of 310 spike trains from 31 single neurons recorded from 21 rats were analyzed. The autocorrelogram of all units were calculated, and only units with less than 0.5% of spike intervals within a 1 ms refractory period were included in the present analysis. We used spike trains only from units having high signal-to-noise ratios (≥5-fold noise level). These units were considered as putative pyramidal cells, based on their wider spike shapes (mean width, >400 *μ*s). Figures 1(e) and 1(f) show a representative unit recorded before and after tianeptine application using AMPA excitation. Spike trains were evoked by either ejecting AMPA or NMDA, using weak or strong ejection currents. For representative recordings (see Supplementary Figures  1–3 in available online at doi: 10.1155/2012/286215). The level of weak excitation (ejection current) was set between 3 and 4 spike/100 ms, whereas strong excitation was set between 6 and 8 spike/100 ms. Intraperitoneal tianeptine application resulted in an enhancement of AMPA-evoked spiking activity, which reached 7–9 spike/100 ms, 30 min after drug administration. Five such spike trains were analyzed from every recording. This group will be referred to as “AMPA after tianeptine.” Accordingly enhanced AMPA sensitivity was induced by a bolus of intraperitoneal tianeptine administration (20 mg/kg, described elsewhere).

**Figure 2:**
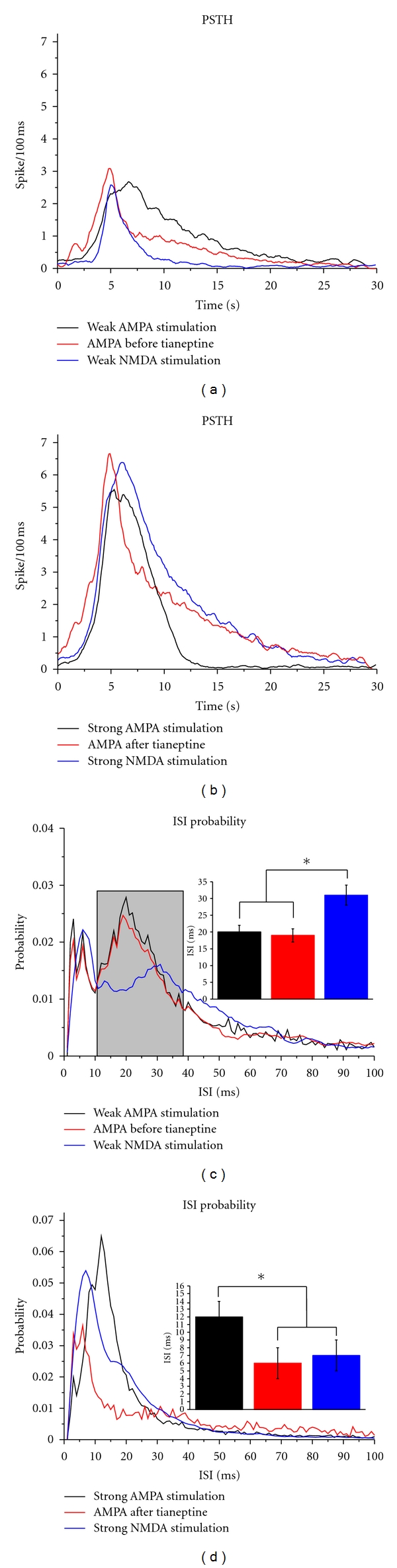
PSTH and ISI probability profiles of different spike trains. There is no difference between the PSTHs of spike trains evoked by weak excitation (a); in contrast, strong AMPA-evoked spiking activity decayed faster than strong NMDA or AMPA after tianeptine spike trains (b). ISI probability distributions of weak excitation evoked spike trains (c). Inset shows the ISI values for the second probability peak (marked with a grey rectangle). ISI probability distributions of strong excitation evoked spike trains (d). Inset shows the ISI values for the maximal probabilities. Note the difference between strong AMPA and AMPA after tianeptine. Colors in the insets correspond to the colors in the main figure. Asterisks denote significant differences at *P* ≤ 0.05.

First, we compared the mean poststimulus time histogram (PSTH) of weak-strong AMPA/NMDA stimulation and AMPA stimulation before and after tianeptine (Figures 2(a) and 2(b)). A one-way repeated measure analysis of variance (ANOVA) with *post hoc* Tukey's test was used for this purpose. We hypothesized that there should be no difference between the weak AMPA stimulation and before tianeptine PSTH data, because no phosphorylation occurred, and the number of evoked spikes is in the same order of magnitude in both groups (the stimulation intensity was comparable). Indeed, there was no difference between weak AMPA, weak NMDA, and AMPA before tianeptine PSTHs (number of spike trains; *n* = 60, 50 and 45, resp.). Similarly, we found no difference between strong AMPA and AMPA after tianeptine PSTHs (*n* = 60 and 45), suggesting that the change in the AMPA gain level is not manifested in the PSTH profile. In contrast, strong AMPA PSTH tended to decay faster than strong NMDA-evoked spike trains (*n* = 50 and 60; *P* ≤ 0.05).

Next, we analyzed the distribution of interspike intervals (ISI) in the evoked spike trains (Figure 2). No significant difference was found in the ISI probability profile of weak AMPA and before tianeptine groups (*n* = 60 and 45). Two probability peaks were seen at around 3–6 ms and 19–21 ms. Weak NMDA-evoked activity resulted in a different ISI probability pattern, also displaying two local maxima (at 3-4 ms and 30-31 ms), although the latter is smaller (marked with a gray rectangle at Figure 2(c); *n* = 50 and 60; *P* ≤ 0.05 compared to weak AMPA and before tianeptine).

Increasing the stimulation strength by applying larger ejection currents resulted in a prominent peak in the ISI probability distribution, suggesting that the spiking activity became more ordered at stronger stimulation. Significant differences were observed between weak AMPA/NMDA (*n* = 60 and 50) and strong AMPA/NMDA, respectively (*P* ≤ 0.05). Furthermore, there was a significant difference between the “strong AMPA” and “AMPA after tianeptine” groups, suggesting that the increase in AMPA sensitivity has an impact on ISI probability (Figure 2(d)). The maximum probability for AMPA after tianeptine was seen at 5–7 ms (*n* = 45). Also, there was difference between strong AMPA and NMDA-evoked activity: the maximum probability was seen at 10–13 ms for strong AMPA, and 6–8 ms for strong NMDA (*n* = 60 and 50; *P* ≤ 0.05).

### 3.1. Temporal Evolution of Spike Trains

**Figure 3:**
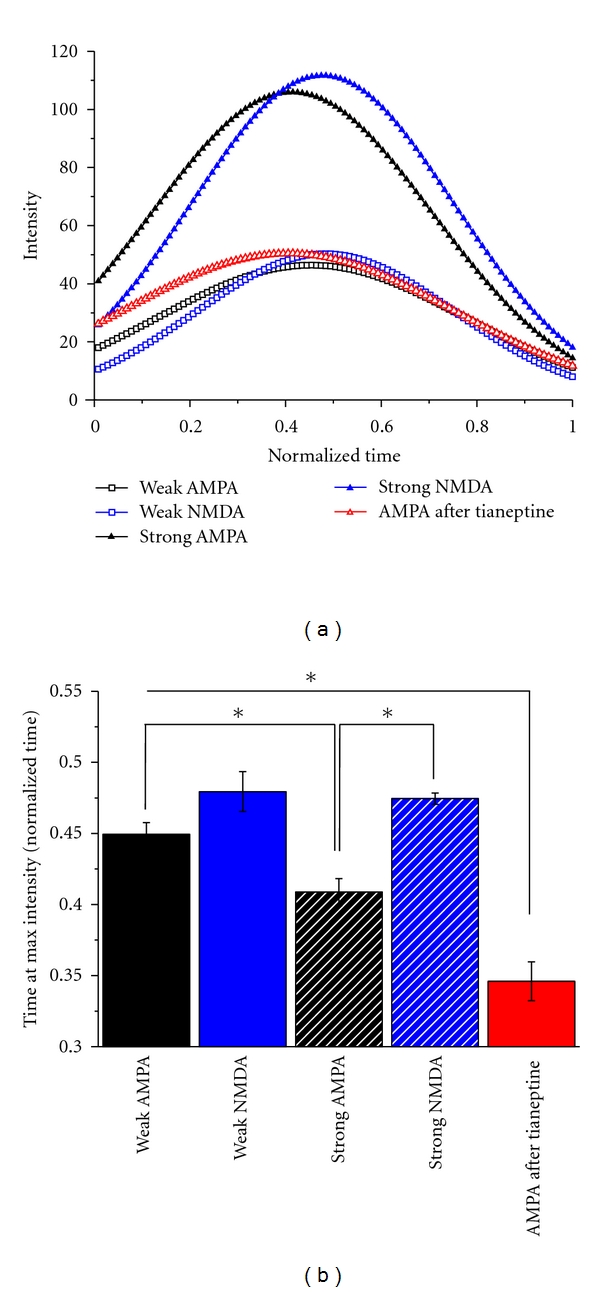
Temporal evolution of spiking dynamics. The time courses of discharge trains were normalized, and a parabola was fitted onto the 1/ISI values (a). Note that the maximal intensity is attained for both AMPA and NMDA excitation regardless of the strength, which happens later than in the AMPA after tianeptine scenario (b). Asterisks denote significant differences at *P* ≤ 0.05.

Next we analyzed the temporal pattern of spiking activity. First, we focused on the temporal evolution of ISIs. A parabola was fitted onto the instantaneous frequency (1/ISI) data (Figure 3(a)). The obtained coefficients are describable by biological terms, namely, the maximal instantaneous frequency exactly provides the position with highest intensity within the spike train (*a*), while *c* corresponds to the dynamism of the change in intensity. We note that the most pronounced difference lies in the time at which maximal intensity is attained (*a*0), which occurs significantly (*P* ≤ 0.05) earlier after application of tianeptine (0.346 ± 0.013) than on the otherwise excited cells (0.472 ± 0.003 for strong NMDA, 0.408 ± 0.009 for strong AMPA, 0.479 ± 0.014 for weak NMDA, and 0.449 ± 0.008 for weak AMPA, resp.). We also detected significant difference between weak AMPA and strong AMPA (*P* ≤ 0.05) as well as strong AMPA and strong NMDA (*P* ≤ 0.05).

## 4. Discussion

AMPA and NMDA receptors (AMPAR and NMDAR, resp.) are the main contributors to excitatory synaptic function, and information is transferred by activation of both receptor types [17, 18]. The relative contribution of these two receptors will affect the postsynaptic transmission and temporal summation, properties which may have important consequences in circuit function. However, posttranslational modifications can alter single-channel properties and postsynaptic function without affecting the ratio of expressed channels. Here, we have investigated whether there is a difference in NMDA or AMPA-evoked spiking patterns of CA1 neurons. We also addressed whether there is a difference in AMPA-evoked firing activity between increasing the gain of previously activated inputs and activating new inputs. By using somatic discharge as an indirect measure of depotentiation, we applied stimuli with similar strengths for either AMPA or NMDA receptors and compared the temporal patterns of evoked spike trains.

AMPA receptors are regarded as rapid activating and rapid desensitizing channels [19, 20], whereas NMDARs have a much slower kinetic [21]. Indeed, we found shorter latency of spiking initiation for AMPA-evoked spike trains (Figure 3(a)) however, ISI values increased quicker compared to NMDA-induced activity. This was more evident when we applied strong stimulation and may be due to the faster desensitization of single-AMPA receptors [19] compared to NMDARs [22]. Strong NMDA activation could maintain a fast firing activation; therefore, ISIs were kept at lower values for a more prolonged time. Increasing the concentration of AMPA/NMDA or increasing the gain of previously activated inputs by tianeptine application may also trigger the activation of local inhibitory circuits, which may have an impact on the ISI profile. Keeping this in mind, it is possible that different inhibitory circuits were activated after applying stronger AMPA than AMPA after tianeptine, which could have differently modified ISI distribution. Indeed, results show that the ISI profile and the time at max intensity was shifted to the left both at strong AMPA and AMPA after tianeptine compared to weak AMPA. It is noteworthy, however, that spike trains after tianeptine application had quicker onsets and more shifted-to-the-left ISI distribution, that the strong AMPA group suggesting either reduced inhibition, or enhanced gain. The former scenario is less likely, because tianeptine was shown to leave the concentration and affinity of GABA-A and GABA-B receptors unaffected [23, 24]. The ratio of the contribution of these receptor subtypes to postsynaptic transmission may underlie specific aspects of synaptic plasticity. For example, the early phase of long-term potentiation (LTP) is thought to selectively enhance AMPA currents and alter the NMDA-to-AMPA ratio [25–28], and during homeostatic synaptic plasticity, AMPA and NMDA currents are scaled up and down proportionally [29]. It is noteworthy that the peaks of ISI probability for strong AMPA (118 ms) and NMDA (53 ms) stimulation correspond to theta- (8-9 Hz) and low-gamma (18-19 Hz) activity, respectively, suggesting that different receptors may be involved in different oscillatory patterns.

Increasing the gain of the cellular input could be achieved by either activating novel receptors (by increasing the amount of NMDA/AMPA), or alternatively, by enhancing the function of already activated inputs. Tianeptine may increase AMPA gain in two overlapping ways: either by enhancing the phosphorylation level of GluA1, as was shown by [14–16], or prompting new AMPA receptor complexes to be trafficked into the synaptic membrane. Both ways could lead to increased AMPA sensitivity, in turn to increased firing rate elicited by activating the previously active inputs (weak AMPA stimulation). Here, we examined the relative contribution of the modified channel properties versus activation of new inputs to the enhanced synaptic strength. There is considerable evidence that GluA1 phosphorylation alters the single-channel properties of the AMPA receptor complex. By using patch clamp techniques, it was shown that phosphorylation of the GluA1 subunit increases AMPAR complex efficiency. Phosphorylation at the PKA site (serine 845) is known to increase the channel opening probability [30] and the peak amplitude of the current [31], while phosphorylation at the PKC/CaMKII site (serine 831) increases the single-channel conductance [32]. Moreover, GluA1 phosphorylation may induce the expression of novel AMPA receptors into the synapse, resulting in higher AMPAR concentration on the cellular surface [33, 34].

In order to induce GluA1 phosphorylation, we applied tianeptine, an antidepressant, which, although transiently, was shown to enhance the phosphorylation level of GluA1 at both the PKA and CaMKII sites [14, 16]. We, however, have used a higher tianeptine concentration (20 mg/kg instead of 10 mg/kg), which may induce a more long-lasting increase of p-GluA1. In keeping with this, we found that after tianeptine treatment, AMPA-evoked spiking with higher probability of small ISIs was compared to strong AMPA stimulation *in vivo*. This may be due to the altered inactivation kinetics of AMPARs. The temporal evolution of spiking dynamics (Figure 3) shows that the relative location of the maximum intensity of discharge activity within a spike train is altered (Figure 3(b)) in the AMPA after tianeptine scenario, suggesting that higher AMPA gain leads to a more sustained rapid discharge activity. In line with this, Banke et al. [30] have shown that phosphorylation of GluA1 at the PKA site does not affect the rate of recovery from desensitization. Similar results were obtained for the CaMK-II site [35], thus phosphorylation of both sites or alternatively higher AMPAR surface concentration may be responsible for the observed effect.

Tianeptine may have other mechanisms of action as well. Although originally it was thought to be a selective serotonin reuptake enhancer, recent studies demonstrated that acute and chronic application of tianeptine does not modify the extracellular concentration of serotonin in freely moving rats [36, 37]. Other data suggest that tianeptine enhances dopaminergic transmission by a yet unknown mechanism [38]. The effects of dopamine in the hippocampus include phosphorylation of GluA1 and certain NMDAR subunits *in vitro* [39] and *in vivo* [40]. We, however, have found that a single bolus of tianeptine injection did not enhance NMDA receptor-mediated responses [15], suggesting that under our experimental conditions, enhancing dopaminergic transmission are not likely to contribute to the findings reported here. Instead, we propose that the observed effects of tianeptine are due to improving neuroplasticity by mainly increasing GluA1 phosphorylation [24].

Both the rate and timing of spiking activity may be important for information coding. Numerous studies have shown that the binned spike counts (rate) encode information [41, 42]. According to the temporal coding hypothesis [43], however, neurons encode information by the exact timing of spikes [12, 44, 45]. Recent reports indicate that the information about the position is coded by both the rate and timing of place-cell activity [46, 47]. In the hippocampus, various physiological states, like sleep [48], and learning [49] can alter GluA1 phosphorylation, and in turn surface AMPAR expression, implying that this phenomenon is dynamically regulated and may have important functions in encoding and processing of memory traces. Recently, experience was shown to be a major factor for transforming a rate code into a more reliable temporal code [47], which, in light of our result, suggests the involvement of physiological GluA1 phosphorylation.

Taken together, our findings further support the role of the dynamic modulation of NMDAR/AMPAR ratio and GluA1 phosphorylation in synaptic plasticity, learning, and memory.

## Supplementary Material

Supplementary Figures: show representative recordings of each recording condition.

## References

[1] Hoffman DA, Sprengel R, Sakmann B (2002). Molecular dissection of hippocampal theta-burst pairing potentiation. *Proceedings of the National Academy of Sciences of the United States of America*.

[2] Jensen V, Kaiser KMM, Borchardt T (2003). A juvenile form of postsynaptic hippocampal long-term potentiation in mice deficient for the AMPA receptor subunit GluR-A. *The Journal of Physiology*.

[3] Malinow R, Malenka RC (2002). AMPA receptor trafficking and synaptic plasticity. *Annual Review of Neuroscience*.

[4] Zamanillo D, Sprengel R, Hvalby O (1999). Importance of AMPA receptors for hippocampal synaptic plasticity but not for spatial learning. *Science*.

[5] Riehle A, Grun S, Diesmann M, Aertsen A (1997). Spike synchronization and rate modulation differentially involved in motor cortical function. *Science*.

[6] Schmitt WB, Arianpour R, Deacon RMJ (2004). The role of hippocampal glutamate receptor-A-dependent synaptic plasticity in conditional learning: the importance of spatiotemporal discontiguity. *Journal of Neuroscience*.

[7] Schmitt WB, Deacon RMJ, Seeburg PH, Rawlins JNP, Bannerman DM (2003). A within-subjects, within-task demonstration of intact spatial reference memory and impaired spatial working memory in glutamate receptor-A-deficient mice. *Journal of Neuroscience*.

[8] Barria A, Muller D, Derkach V, Griffith LC, Soderling TR (1997). Regulatory phosphorylation of AMPA-type glutamate receptors by CaM-KII during long-term potentiation. *Science*.

[9] Mammen AL, Kameyama K, Roche KW, Huganir RL (1997). Phosphorylation of the *α*-amino-3-hydroxy-5-methylisoxazole-4-propionic Acid receptor GluR1 subunit by calcium/calmodulin-dependent kinase II. *The Journal of Biological Chemistry*.

[10] Buzsaki G, Chrobak JJ (1995). Temporal structure in spatially organized neuronal ensembles: a role for interneuronal networks. *Current Opinion in Neurobiology*.

[11] Gray CM, Singer W (1989). Stimulus-specific neuronal oscillations in orientation columns of cat visual cortex. *Proceedings of the National Academy of Sciences of the United States of America*.

[12] Hopfield JJ (1995). Pattern recognition computation using action potential timing for stimulus representation. *Nature*.

[13] Vaadia E, Haalman I, Abeles M (1995). Dynamics of neuronal interactions in monkey cortex in relation to behavioural events. *Nature*.

[14] Qi H, Mailliet F, Spedding M (2009). Antidepressants reverse the attenuation of the neurotrophic MEK/MAPK cascade in frontal cortex by elevated platform stress; reversal of effects on LTP is associated with GluA1 phosphorylation. *Neuropharmacology*.

[15] Szegedi V, Juhasz G, Zhang X (2011). Tianeptine potentiates AMPA receptors by activating CaMKII and PKA via the p38, p42/44 MAPK and JNK pathways. *Neurochemistry International*.

[16] Svenningsson P, Bateup H, Qi H (2007). Involvement of AMPA receptor phosphorylation in antidepressant actions with special reference to tianeptine. *European Journal of Neuroscience*.

[17] Daw NW, Stein PSG, Fox K (1993). The role of NMDA receptors in information processing. *Annual Review of Neuroscience*.

[18] Rivadulla C, Sharma J, Sur M (2001). Specific roles of NMDA and AMPA receptors in direction-selective and spatial phase-selective responses in visual cortex. *Journal of Neuroscience*.

[19] Colquhoun D, Jonas P, Sakmann B (1992). Action of brief pulses of glutamate on AMPA/kainate receptors in patches from different neurones of rat hippocampal slices. *The Journal of Physiology*.

[20] Hestrin S (1992). Activation and desensitization of glutamate-activated channels mediating fast excitatory synaptic currents in the visual cortex. *Neuron*.

[21] McBain CJ, Mayer ML (1994). N-methyl-D-aspartic acid receptor structure and function. *Physiological Reviews*.

[22] Kussius CL, Kaur N, Popescu GK (2009). Pregnanolone sulfate promotes desensitization of activated NMDA receptors. *Journal of Neuroscience*.

[23] Kato G, Weitsch AF (1988). Neurochemical profile of tianeptine, a new antidepressant drug. *Clinical Neuropharmacology*.

[24] McEwen BS, Chattarji S, Diamond DM (2010). The neurobiological properties of tianeptine (Stablon): from monoamine hypothesis to glutamatergic modulation. *Molecular Psychiatry*.

[25] Kauer JA, Malenka RC, Nicoll RA (1988). A persistent postsynaptic modification mediates long-term potentiation in the hippocampus. *Neuron*.

[26] Liao D, Hessler NA, Malinow R (1995). Activation of postsynaptically silent synapses during pairing-induced LTP in CA1 region of hippocampal slice. *Nature*.

[27] Lu WY, Man HY, Ju W, Trimble WS, MacDonald JF, Wang YT (2001). Activation of synaptic NMDA receptors induces membrane insertion of new AMPA receptors and LTP in cultured hippocampal neurons. *Neuron*.

[28] Watt AJ, Sjostrom PJ, Hausser M, Nelson SB, Turrigiano GG (2004). A proportional but slower NMDA potentiation follows AMPA potentiation in LTP. *Nature Neuroscience*.

[29] Watt AJ, van Rossum MCW, MacLeod KM, Nelson SB, Turrigiano GG (2000). Activity coregulates quantal AMPA and NMDA currents at neocortical synapses. *Neuron*.

[30] Banke TG, Bowie D, Lee HK, Huganir RL, Schousboe A, Traynelis SF (2000). Control of GluR1 AMPA receptor function by cAMP-dependent protein kinase. *Journal of Neuroscience*.

[31] Roche KW, O’Brien RJ, Mammen AL, Bernhardt J, Huganir RL (1996). Characterization of multiple phosphorylation sites on the AMPA receptor GluR1 subunit. *Neuron*.

[32] Derkach VA (2003). Silence analysis of AMPA receptor mutated at the CAM-kinase II phosphorylation site. *Biophysical Journal*.

[33] Hayashi Y, Shi SH, Esteban JA, Piccini A, Poncer JC, Malinow R (2000). Driving AMPA receptors into synapses by LTP and CaMKII: requirement for GluR1 and PDZ domain interaction. *Science*.

[34] Lee HK, Barbarosie M, Kameyama K, Bear MF, Huganir RL (2000). Regulation of distinct AMPA receptor phosphorylation sites during bidirectional synaptic plasticity. *Nature*.

[35] Derkach V, Barria A, Soderling TR (1999). Ca^2+^/calmodulin-kinase II enhances channel conductance of *α*-amino-3-hydroxy-5-methyl-4-isoxazolepropionate type glutamate receptors. *Proceedings of the National Academy of Sciences of the United States of America*.

[36] Malagie I, Deslandes A, Gardier AM (2000). Effects of acute and chronic tianeptine administration on serotonin outflow in rats: comparison with paroxetine by using in vivo microdialysis. *European Journal of Pharmacology*.

[37] Pineyro G, Deveault L, de Montigny C, Blier P (1995). Effect of prolonged administration of tianeptine on 5-HT neurotransmission: an electrophysiological study in the rat hippocampus and dorsal raphe. *Naunyn-Schmiedeberg’s Archives of Pharmacology*.

[38] Invernizzi R, Pozzi L, Garattini S, Samanin R (1992). Tianeptine increases the extracellular concentrations of dopamine in the nucleus accumbens by a serotonin-independent mechanism. *Neuropharmacology*.

[39] Sarantis K, Matsokis N, Angelatou F (2009). Synergistic interactions of dopamine D1 and glutamate NMDA receptors in rat hippocampus and prefrontal cortex: involvement of ERK1/2 signaling. *Neuroscience*.

[40] Sarantis K, Antoniou K, Matsokis N, Angelatou F (2012). Exposure to novel environment is characterized by an interaction of D1/NMDA receptors underlined by phosphorylation of the NMDA and AMPA receptor subunits and activation of ERK1/2 signaling, leading to epigenetic changes and gene expression in rat hippocampus. *Neurochemistry International*.

[41] O’Keefe J, Dostrovsky J (1971). The hippocampus as a spatial map. Preliminary evidence from unit activity in the freely-moving rat. *Brain Research*.

[42] Wilson MA, McNaughton BL (1993). Dynamics of the hippocampal ensemble code for space. *Science*.

[43] Singer W (1993). Synchronization of cortical activity and its putative role in information processing and learning. *Annual Review of Physiology*.

[44] O’Keefe J, Recce ML (1993). Phase relationship between hippocampal place units and the EEG theta rhythm. *Hippocampus*.

[45] Skaggs WE, McNaughton BL, Wilson MA, Barnes CA (1996). Theta phase precession in hippocampal neuronal populations and the compression of temporal sequences. *Hippocampus*.

[46] Harris KD, Henze DA, Hirase H (2002). Spike train dynamics predicts theta-related phase precession in hippocampal pyramidal cells. *Nature*.

[47] Mehta MR, Lee AK, Wilson MA (2002). Role of experience and oscillations in transforming a rate code into a temporal code. *Nature*.

[48] Hagewoud R, Havekes R, Novati A, Keijser JN, van der Zee EA, Meerlo P (2010). Sleep deprivation impairs spatial working memory and reduces hippocampal AMPA receptor phosphorylation. *Journal of Sleep Research*.

[49] Uslaner JM, Parmentier-Batteur S, Flick RB (2009). Dose-dependent effect of CDPPB, the mGluR5 positive allosteric modulator, on recognition memory is associated with GluR1 and CREB phosphorylation in the prefrontal cortex and hippocampus. *Neuropharmacology*.

