# Supplementary figures and images for: GluA1 Phosphorylation Alters Evoked Firing Pattern In Vivo
Source: Neural Plast. 2012 Apr 9;2012:286215. doi: 10.1155/2012/286215 (PMC3337492; doi:10.1155/2012/286215)

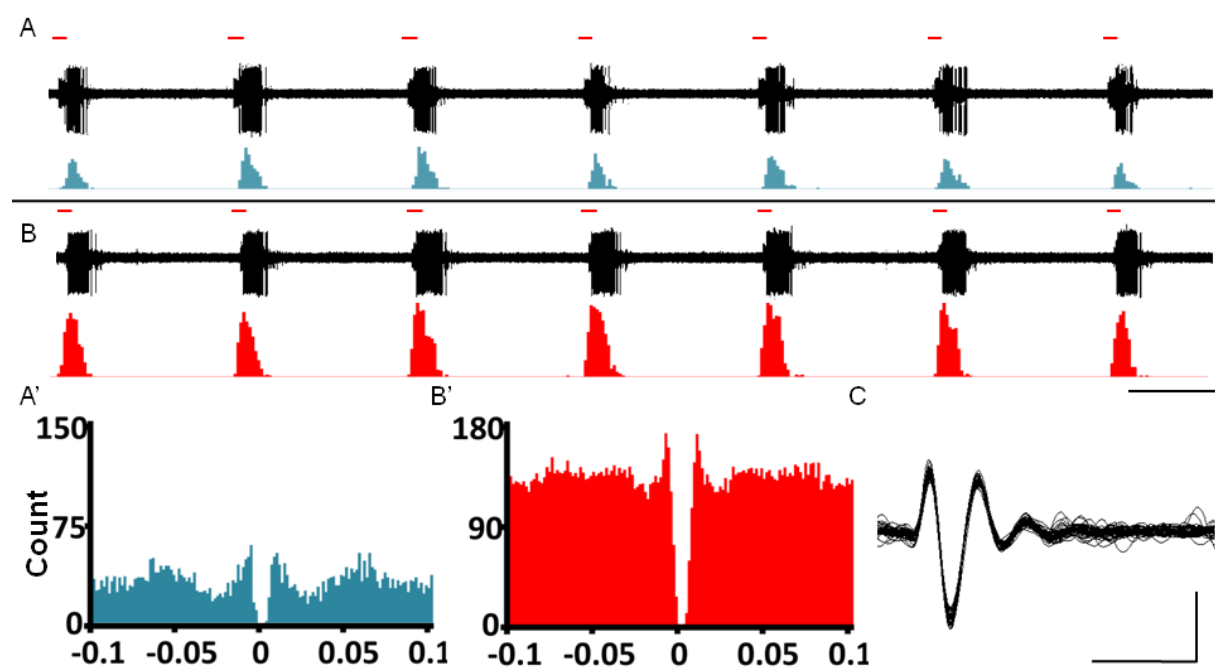

Supplementary Figure 1.

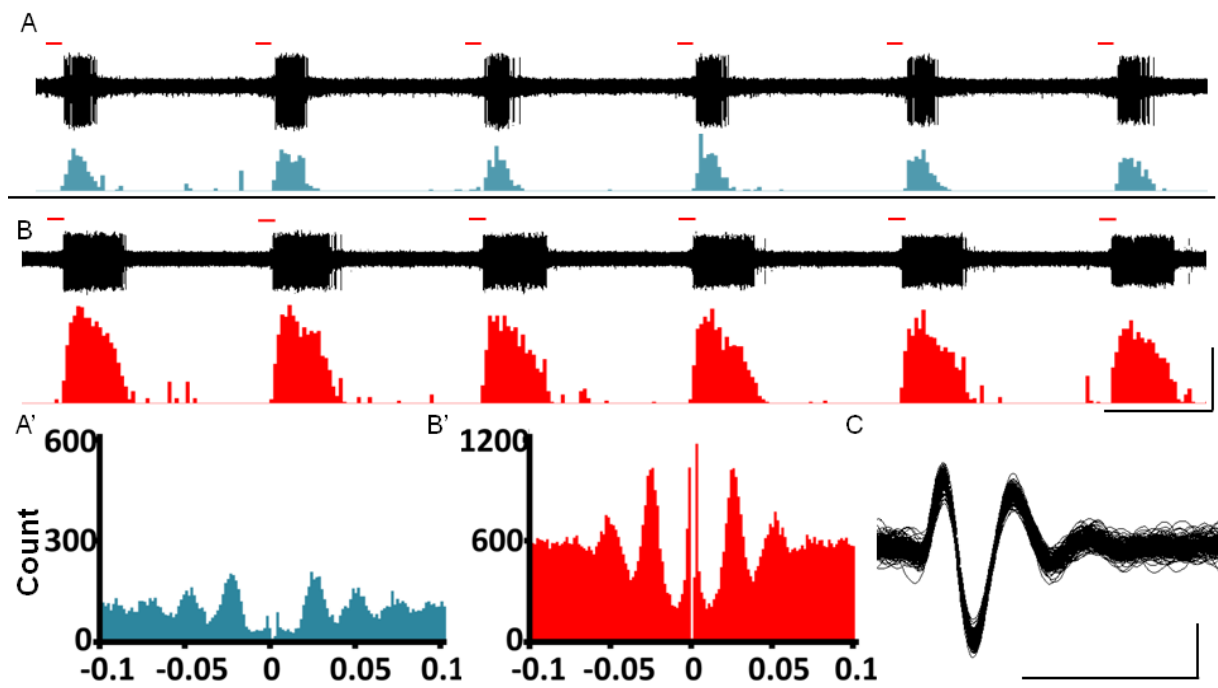

Supplementary Figure 2.

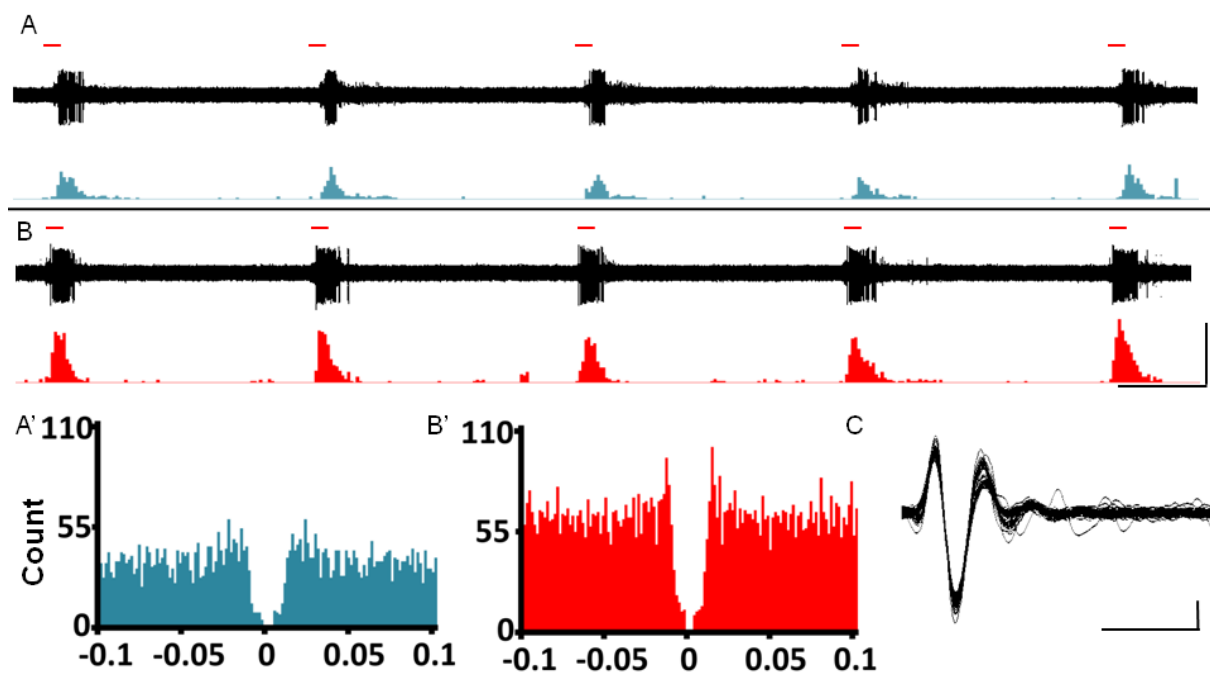

Supplementary Figure 3.

Supplement: Supplementary file 1 — Supplementary Figures: show representative recordings of each recording condition. [file 286215.f1.pdf]
